# Sequence-Activity Relationships and Integrated *In Silico* and *In Vitro* Mechanistic Study of the Antimicrobial Peptide PEP‑4 against *Staphylococcus aureus*

**DOI:** 10.1021/acsomega.6c03955

**Published:** 2026-10-16

**Authors:** Isabel Ladeira Pereira, Isadora Leitzke Guidotti, Danillo de Oliveira Della Senta, Isadora Cosenza Vieira da Silva, Fabiana Kommling Seixas, Leandro Farias Rodrigues, Luciano da Silva Pinto, Daiane Hartwig, Frederico Schmitt Kremer

**Affiliations:** † Centrode Desenvolvimento Tecnológico, 37902 Universidade Federal de Pelotas, Campus Universitário Capão do Leão, 96160-000 Capãodo Leão, Rio Grande do Sul, Brazil; ‡ Faculdade de Enfermagem, Universidade Federal de Pelotas, 96010-610 Pelotas, Rio Grande do Sul, Brazil; § Departamentode Microbiologia e Parasitologia, Instituto de Biologia, Universidade Federal de Pelotas, Campus Universitário Capão do Leão, 96160-000 Capãodo Leão, Rio Grande do Sul, Brazil

## Abstract

The rise of antimicrobial resistance among ESKAPE pathogens demands novel therapeutic strategies. Here, we evaluate a panel of five synthetic peptides through integrated *in vitro* and *in silico* analyses, highlighting PEP-4 as a promising antibacterial lead peptide. Our findings demonstrate the key role of C-terminal hydrophobic patterning in antimicrobial efficacy. PEP-4 exhibits strong antibacterial activity (MIC 15.6–62.5 μg mL^–1^, corresponding to ∼10.5–41.9 μM) and consistently shows bactericidal effects against both Gram-positive and Gram-negative strains. Molecular dynamics simulations show sustained, charge-selective interaction of PEP-4 with an anionic *S. aureus*-like bilayer and only intermittent association with a zwitterionic mammalian model, supporting electrostatically driven membrane recognition as the initial step of its mechanism; no bilayer destabilization was observed in either system over the simulated time scale. Additionally, molecular docking suggests potential secondary intracellular protein targets. Notably, PEP-4 retains activity in the presence of lysyl-phospholipid-mediated resistance in *Staphylococcus aureus*, yet exhibits only transient interactions with zwitterionic mammalian membranes. This selective membrane binding is consistent with low cytotoxicity to humans, corroborated by other *in silico* predictions. It suggests a potential therapeutic window, pending experimental validation, supporting PEP-4 as a promising peptide scaffold for next-generation antimicrobial design.

## Graphical abstract

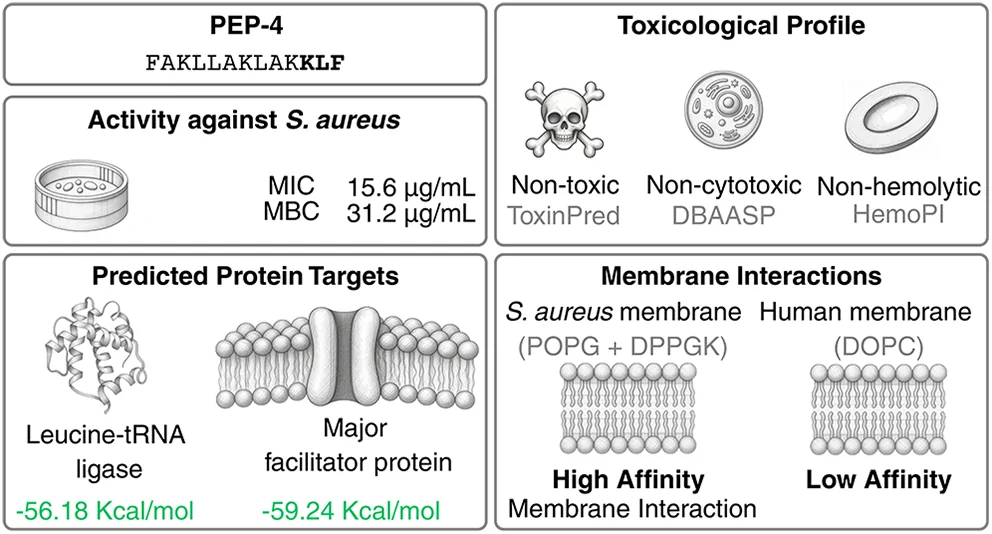

## Introduction

The rapid emergence and global dissemination of antimicrobial resistance (AMR) constitute one of the most pressing challenges for modern medicine. Recent estimates indicate that bacterial AMR is associated with millions of deaths annually, with projections suggesting a substantial increase in the coming decades if current trends persist.^1^ Beyond clinical settings, AMR represents a complex concern within the One Health paradigm,^2,3^ where the widespread use and misuse of antimicrobials across human, animal, and environmental domains drive the selection and global spread of resistant microorganisms and genes.^4^

This burden is particularly pronounced in healthcare settings, where infections caused by multidrug-resistant organisms significantly increase morbidity, mortality, and healthcare costs.^5^ Among these, the ESKAPE pathogens (*Enterococcus faecium*, *Staphylococcus aureus*, *Klebsiella pneumoniae*, *Acinetobacter baumannii*, *Pseudomonas aeruginosa*, and *Enterobacter* spp.) represent a critical group of opportunistic bacteria responsible for the majority of nosocomial infections worldwide.^6^ These pathogens possess a high capacity to acquire and disseminate resistance determinants, effectively bypassing conventional antibiotics and severely limiting available therapeutic options.^7,8^

Despite the urgency of the AMR crisis, the development of new antibiotics has progressed too slowly to keep pace with the rapid emergence of resistant pathogens, largely because of economic, regulatory, and scientific challenges. This growing therapeutic gap necessitates exploring alternative antimicrobial strategies that operate through mechanisms less prone to resistance development.^9,10^ In this context, antimicrobial peptides (AMPs) have emerged as promising candidates.^11^ These AMPs, which are key components of innate immunity, typically exert their activity through membrane-targeting mechanisms involving electrostatic interactions with negatively charged bacterial membranes, followed by hydrophobic insertion and membrane destabilization. This mode of action reduces the likelihood of resistance development compared with conventional antibiotics that target specific intracellular pathways.^12^

Building on this rationale, the present study evaluates the antimicrobial activity of five synthetic peptides originally designed using TACaPE, an in-house generative framework for peptide design (https://github.com/omixlab/anticancer-peptide). We systematically assess their efficacy against the full panel of ESKAPE pathogens and investigate their mechanism of action through an integrated computational–experimental approach. Specifically, we combine physicochemical characterization, *in vitro* antimicrobial assays, and long-time-scale molecular dynamics simulations to elucidate peptide–membrane interactions at atomic resolution. Furthermore, we employ reverse molecular docking as an exploratory strategy to investigate potential intracellular mechanisms of action and identify protein interaction partners that may warrant future investigation, in which translocated AMPs may amplify their efficacy by engaging cytoplasmic targets secondary to primary membrane disruption. In this study, we employed susceptible ESKAPE reference strains as standardized models to establish baseline antimicrobial activity and evaluate structure–activity relationships prior to future evaluations against resistant clinical isolates.

## Materials and Methods

### Computational Peptide Design

We designed the five peptides investigated in this study by using the TACaPE framework. Briefly, TACaPE is an autoregressive generative model that iteratively predicts the next amino acid in a sequence by sampling a learned probability distribution conditioned on the preceding amino acids. We initiated generation using a ″begin of sentence″ (BOS) token and trained the model using a deduplicated data set. To guide sequence selection, the framework prioritizes candidates with optimal physicochemical properties, such as cationic character and amphipathicity, and employs a classification probability threshold of 0.5 to filter the sequences.

### Peptides, Reagents, and Bacterial Strains

Genone (Brazil) synthesized the five peptides shown in Table 1 via solid-phase synthesis with >95% purity, as confirmed by HPLC. Both theoretical and experimental molecular weights were shown to verify peptide identity. We selected these synthetic peptides as representative variants that differ primarily in the C-terminal region, enabling an experimental evaluation of how terminal hydrophobic residues influence antibacterial activity.

**Table 1:** Amino Acid Sequences of the Synthetic Peptides Evaluated in This Study

name	sequence	molecular weight (theoretical)	molecular weight (experimental)
PEP-1	FAKLLAKLAKKIL	1456.92	1456.9
PEP-2	FAKLLAKFAKKLA	1448.86	1448.84
PEP-3	FAKLLAKLAKKFA	1448.86	1448.84
PEP-4	FAKLLAKLAKKLF	1490.94	1490.92
PEP-5	FAKLLAKFAKKAL	1448.86	1448.84

### *In Silico* Characterization

We calculated the physicochemical properties of the five synthetic peptides using the HeliQuest web server^13^ to determine hydrophobicity (H), hydrophobic moment (μH), and net charge, and to generate helical wheel projections. We predicted potential antimicrobial activity using DBAASP^14^ and AMPScanner,^15^ hemolytic propensity using HemoPI,^16^ and toxicity using ToxinPred.^17^ Finally, we predicted allergenicity utilizing AllerCatPro.^18^

### Bacterial Strains and Culture Conditions

For the antibacterial assays, we used ESKAPE reference strains: *Enterococcus faecium* NCTC 12202, *Staphylococcus aureus* ATCC 12600, *Klebsiella pneumoniae* ATCC 700603, *Acinetobacter baumannii* ATCC 19606, *Pseudomonas aeruginosa* ATCC 27853, and *Klebsiella aerogenes* ATCC 13048 (formerly *Enterobacter aerogenes*). We stored bacterial strains at −20 °C in a cryoprotective solution containing 5% (w/v) nonfat dry milk and 10% (v/v) glycerol (Sigma-Aldrich, Co.). Before each experiment, we reactivated strains in Brain Heart Infusion (BHI) broth (Kasvi, BRA). We subsequently cultured reactivated cultures onto selective agar plates: Gram-negative bacteria (*A. baumannii*, *K. pneumoniae*, *K. aerogenes*, and *P. aeruginosa*) on MacConkey agar plates (Kasvi, BRA); *S. aureus* on Mannitol Salt Agar (Kasvi, BRA); and *E. faecium* on Mitis Salivarius agar (Synth, BRA). We incubated all cultures at 37 °C for 24 h under aerobic conditions.

### Minimum Inhibitory Concentration (MIC)

We evaluated five synthetic peptides in the antibacterial assay. We prepared peptide stock solutions (1 mg mL^–1^) in water for injection containing 5% dimethyl sulfoxide (DMSO). We prepared serial 2-fold dilutions in broth to obtain final peptide concentrations ranging from 0.48 to 500 μg mL^–1^. We determined the minimum inhibitory concentration (MIC) using the broth microdilution method in sterile 96-well polystyrene microplates as established by Clinical and Laboratory Standards Institute guidelines.^19^ We subcultured bacterial strains on BHI agar, suspended freshly grown cultures in the exponential growth phase in sterile saline solution (0.9% NaCl), and adjusted the suspension to the 0.5 McFarland standard (approximately 1.5 × 10^8^ CFU mL^–1^). We then diluted the bacterial suspension in BHI broth to achieve a final inoculum concentration of 5 × 10^5^ CFU mL^–1^ per well. Each well received the appropriate concentration of peptide and bacterial inoculum. Test controls consisted of BHI broth alone (medium control) and BHI broth supplemented with bacterial inoculum (growth control). Sterility controls containing only the synthetic peptides diluted in broth were also included. We performed all assays in technical triplicate and repeated them in at least three independent experiments. After incubation at 37 °C for 18 h, we added 20 μL of 0.2% (w/v) resazurin solution (Sigma-Aldrich) to each well of the microtiter plate. Following incubation at 37 °C for 1 h, we assessed bacterial metabolic activity by observing a color change from blue to pink.^20^

### Minimum Bactericidal Concentration (MBC)

We determined the minimum bactericidal concentration (MBC) from the MIC assay results. After resazurin staining, we collected aliquots from wells showing no detectable metabolic activity and from adjacent wells containing peptide concentrations above the MIC. We plated these aliquots onto selective agar appropriate for each bacterial species and incubated them at 37 °C for 18 h. We interpreted the absence of visible bacterial growth on agar plates as indicative of bactericidal activity, whereas the presence of bacterial growth indicated a bacteriostatic effect.

### Molecular Dynamics

Because standard *in silico* tools could not reliably differentiate the activity profiles of the synthetic peptides, empirical screening was performed to identify the lead peptide. Following its selection and based on its superior *in vitro* bactericidal performance, resource-intensive molecular dynamics simulations and reverse docking were allocated exclusively to PEP-4. We constructed initial membrane-peptide systems using the CHARMM-GUI Membrane Builder.^21^ We modeled the bacterial membrane as a binary lipid bilayer with a total of 76 POPG and 26 DPPGK molecules (approximately a 3:1 ratio), representing the lipid composition of *Staphylococcus aureus*.^22^ We modeled the mammalian membrane as a pure DOPC bilayer containing 102 molecules. We initially positioned PEP-4 in the bulk solvent phase, 4 nm away from the upper lipid leaflet, to prevent biased initial contacts during system equilibration.

We performed all simulations in GROMACS version 2026.0 ^23^ using the CHARMM36m all-atom force field.^24^ We solvated systems using the TIP3P water model and neutralized them with 0.15 M NaCl to replicate the physiological ionic strength. We performed energy minimization using a steep, progressively relaxing schedule until the maximum force fell below 1000 kJ/mol/nm. We equilibrated systems using the standard six-step CHARMM-GUI protocol, progressively relaxing harmonic position restraints on the protein backbone, side chains, and lipid headgroups across the NVT and NPT ensembles.

Production molecular dynamics trajectories consisted of three independent 1 μs replicates for both bacterial membrane model (POPG/DPPGK) and mammalian membrane model (DOPC), resulting in a total simulation time of 6 μs. All production simulations used a 2 fs time step. The temperature was maintained at 303.15 K using the Nosé-Hoover thermostat (coupling time constant = 1.0 ps), applied independently to the protein–lipid and solvent–ion groups. Pressure was maintained at 1.0 bar using the Parrinello–Rahman barostat under semi-isotropic coupling (compressibility = 4.5 × 10^–5^ bar^–1^, time constant = 5.0 ps), allowing independent fluctuation of the XY plane relative to the *Z*-axis. The DOPC bilayer was selected as a simplified, cholesterol-free zwitterionic mammalian membrane model (DOPC) to evaluate baseline membrane selectivity. The intentional absence of cholesterol increases membrane fluidity and susceptibility to peptide-induced perturbation, thereby representing a conservative scenario for assessing potential mammalian membrane disruption.

Short-range van der Waals interactions were calculated using a force-switch modifier that decays to zero between 1.0 and 1.2 nm. Long-range electrostatics were computed via the particle mesh Ewald (PME) algorithm with a 0.16 nm grid spacing and a 1.2 nm real-space cutoff. All covalent bonds involving hydrogen atoms were constrained by using the LINCS algorithm.

We applied periodic boundary conditions (PBCs) in all spatial dimensions and saved trajectory coordinates every 100 ps. Before structural analysis, we processed trajectories to correct for PBC artifacts and dynamically center them on the lipid bilayer. We computed quantitative metrics, including peptide-membrane minimum distance (gmx mindist), interfacial atomic contacts (threshold <0.35 nm), *z*-axis mass density profiles (gmx density), and area per lipid (APL) derived from lateral box vectors (gmx energy), using native GROMACS utilities.

### Reverse Docking to *Staphylococcus aureus* Proteome

We performed reverse molecular docking against the predicted 3D structures of all proteins in *S. aureus* as predicted by AlphaFold^25^ and available in the AlphaFold Database.^26^ We carried out molecular docking with AutoDock CrankPeptide (ADCP), using the prepare_ligand and prepare_receptor scripts from the ADFR package for ligand and receptor file preparation, respectively.^27^

## Results

Computational analysis of the five synthetic peptides (PEP-1 through PEP-5), as shown in Table 2, predicted uniform antibacterial activity across all sequences using AMPScanner, despite sequence variation. *In silico* toxicity prediction via DBAASP and ToxinPred classified all synthetic peptides as noncytotoxic and nontoxic. HemoPI regression models predicted all sequences as nonhemolytic, yielding HC50 values ranging from 234.590 μM for PEP-4 to 471.076 μM for PEP-3.

**Table 2:** *In Silico* Prediction of Antimicrobial Activity, Cytotoxicity, and Hemolytic Propensity for the Synthetic Peptides

	DBAASP			HemoPI regression			
peptide	cytotoxicity	score	AMPScanner	HC_50_ (μM)	prediction	toxic (ToxinPred)	allergenicity (AllerCatPro)
PEP-1	not active	AMP	AMP	283.761	nonhemolytic	nontoxic	non-allergenic
PEP-2	not active	AMP	AMP	418.696	nonhemolytic	nontoxic	non-allergenic
PEP-3	not active	AMP	AMP	471.076	nonhemolytic	nontoxic	non-allergenic
PEP-4	not active	AMP	AMP	234.590	nonhemolytic	nontoxic	non-allergenic
PEP-5	not active	AMP	AMP	442.497	nonhemolytic	nontoxic	non-allergenic

Physicochemical characterization, presented in Table 3, revealed a conserved core structural profile across all five synthetic peptides. Every sequence possessed an identical net charge of 4.00, an isoelectric point of 11.15, an amphiphilicity index of 1.13, an angle subtended by hydrophobic residues of 230.00, and a zero propensity for aggregation. Structural variations were observed in the specific hydrophobicity and membrane insertion metrics. Normalized hydrophobic moment values ranged from 1.80 (PEP-5) to 1.93 (PEP-3). Normalized hydrophobicity was negative across all sequences, with PEP-4 exhibiting the lowest value of −0.79. Predicted membrane penetration depths ranged narrowly from 13 to 14, while insertion tilt angles ranged from 73 in PEP-3 to 86 in PEP-1.

**Table 3:** Physicochemical Characterization and Predicted Secondary Structural Parameters of the Synthetic Peptides^a^

peptide	NHM	NH	NC	IP	PD	TA	DCP	LM	PA	ASHR	AI	PPPII
PEP-1	1.89	–0.74	4.00	11.15	13	86	0.23	0.34	0.00	230.00	1.13	0.89
PEP-2	1.92	–0.65	4.00	11.15	14	76	0.14	0.31	0.00	230.00	1.13	0.90
PEP-3	1.93	–0.65	4.00	11.15	14	73	0.14	0.33	0.00	230.00	1.13	0.90
PEP-4	1.91	–0.79	4.00	11.15	13	80	0.19	0.34	0.00	230.00	1.13	0.89
PEP-5	1.80	–0.65	4.00	11.15	13	83	0.14	0.31	0.00	230.00	1.13	0.90

a NHM = normalized hydrophobic moment; NH = normalized hydrophobicity; NC= net charge; IP = isoelectric point; PD = penetration depth; TA = tilt angle; DCP = disordered conformation propensity; LM = linear moment; PA = propensity to aggregation; ASHR = angle subtended by the hydrophobic residues; AI = amphiphilicity index; and PPPII = propensity to PPII coil.

The *in vitro* antibacterial activity of the synthetic peptides against ESKAPE reference strains is summarized in Table 4. Overall, all synthetic peptides exhibited antibacterial activity against at least some of the tested microorganisms, with MIC values ranging from 15.6 to 500 μg mL^–‑1^.

**Table 4:** Minimum Inhibitory Concentration (MIC) and Minimum Bactericidal Concentration (MBC) of Synthetic Peptides against ESKAPE Reference Strains^a^

		peptide				
ESKAPE reference strain		PEP-1	PEP-2	PEP-3	PEP-4	PEP-5
*E. faecium* NCTC 12202	MIC (μg mL^–1^)	62.5	250	125	31.2	500
	MBC (μg mL^–1^)	125	500	250	62.5	500
*S. aureus* ATCC 12600	MIC (μg mL^–1^)	31.2	500	125	15.6	250
	MBC (μg mL^–1^)	125	>500	250	31.2	500
*K. pneumoniae* ATCC 700603	MIC (μg mL^–1^)	125	500	500	62.5	500
	MBC (μg mL^–1^)	250	500	500	62.5	500
*A. baumannii* ATCC 19606	MIC (μg mL^–1^)	62.5	250	500	62.5	500
	MBC (μg mL^–1^)	62.5	500	500	62.5	500
*P. aeruginosa* ATCC 27853	MIC (μg mL^–1^)	62.5	250	250	62.5	250
	MBC (μg mL^–1^)	62.5	500	250	62.5	250
*K. aerogenes* ATCC 13048	MIC (μg mL^–1^)	125	250	500	62.5	>500
	MBC (μg mL^–1^)	125	500	500	62.5	>500

a Values are expressed in μg mL^–1^.

The Gram-positive strains *Enterococcus faecium* NCTC 12202 and *Staphylococcus aureus* ATCC 12600 were particularly susceptible to the synthetic peptides. All evaluated synthetic peptides exhibited detectable bactericidal activity against *E. faecium*, although with markedly different potencies, as reflected by their MIC and MBC values. In contrast, although *S. aureus* was also susceptible to all synthetic peptides, PEP-2 displayed exclusively bacteriostatic activity, with an MIC of 500 μg mL^–1^ and no detectable bactericidal effect within the tested concentration range. Among the tested synthetic peptides, PEP-4 exhibited the lowest MIC values, indicating superior antibacterial activity against Gram-positive strains, with the lowest MICs against *E. faecium* (31.2 μg mL^–1^) and *S. aureus* (15.6 μg mL^–1^). The MBC values for PEP-4 were 2× MIC, indicating bactericidal activity against *E. faecium* (62.5 μg mL^–1^) and *S. aureus* (31.2 μg mL^–1^).

Distinct susceptibility profiles were observed among the Gram-negative strains *K. pneumoniae* ATCC 700603, *A. baumannii* ATCC 19606, *P. aeruginosa* ATCC 27853, and *K. aerogenes* ATCC 13048 when exposed to the synthetic peptides. Among the evaluated synthetic peptides, PEP-4 exhibited the highest antibacterial activity, with the lowest MIC values across all tested species. Notably, PEP-4 showed both inhibitory and bactericidal activity against *K. pneumoniae*, *A. baumannii*, *P. aeruginosa*, and *K. aerogenes*, with MBC values equal to the corresponding MIC (62.5 μg mL^–1^). In contrast, PEP-5 showed no inhibitory or bactericidal activity against *K. aerogenes*, indicating no antibacterial effect against this species. For the remaining strains, PEP-5 exhibited high MIC values (500 μg mL^–1^) against most species, except for *S. aureus* and *P. aeruginosa*, for which lower MIC values (250 μg mL^–1^) were observed.

Regarding PEP-1, identical MIC values of 62.5 μg mL^–1^ were observed against *P. aeruginosa* and *A. baumannii*, with MBC values equal to the MIC, indicating bactericidal activity for both species. Higher MIC values (125 μg mL^–1^) were detected for *K. pneumoniae* and *K. aerogenes*. However, while this concentration was bactericidal for *K. aerogenes*, bactericidal activity against *K. pneumoniae* was observed only at 250 μg mL^–1^. In addition, PEP-1 was active against all ESKAPE reference strains, including the Gram-positive *E. faecium* and *S. aureus*, with MIC values of 62.5 μg mL^–1^ and 31.2 μg mL^–1^, respectively, and MBC values of 125 μg mL^–1^ for both species.

*In silico* reverse docking against the *S. aureus* proteome identified two candidate interaction partners for PEP-4, consistent with the membrane association observed in molecular dynamics. The highest-ranked interaction, based on the docking score, was identified for the membrane-embedded Major Facilitator Superfamily (MFS) transporter (Q2G0C4; Δ*G* = −59.24 kcal/mol) and is shown in Figure 1; other hits are listed in Table 5. Evaluation of the cytosolic space identified Leucine-tRNA ligase (Q2FXH2; Δ*G* = −56.18 kcal/mol) as the highest-affinity intracellular target. Analysis of the peptide–protein interfaces (Table 6 and Figure 2) revealed distinct interaction profiles between the peptide and the two *S. aureus* targets. The Q2G0C4MFS transporter interface was enriched in hydrophobic residues (Ala, Ile, Leu, Met, Val, and Phe) and contained only a few charged residues, indicating a predominantly membrane-associated interaction surface. In contrast, the Q2FXH2 interface contained a balanced distribution of hydrophobic, polar, and charged residues, including a cluster of aromatic and positively charged residues (Tyr27, Lys28, Arg29, Trp32, Phe34, and Lys35) that may constitute a peptide-binding hotspot.

**Figure 1:**
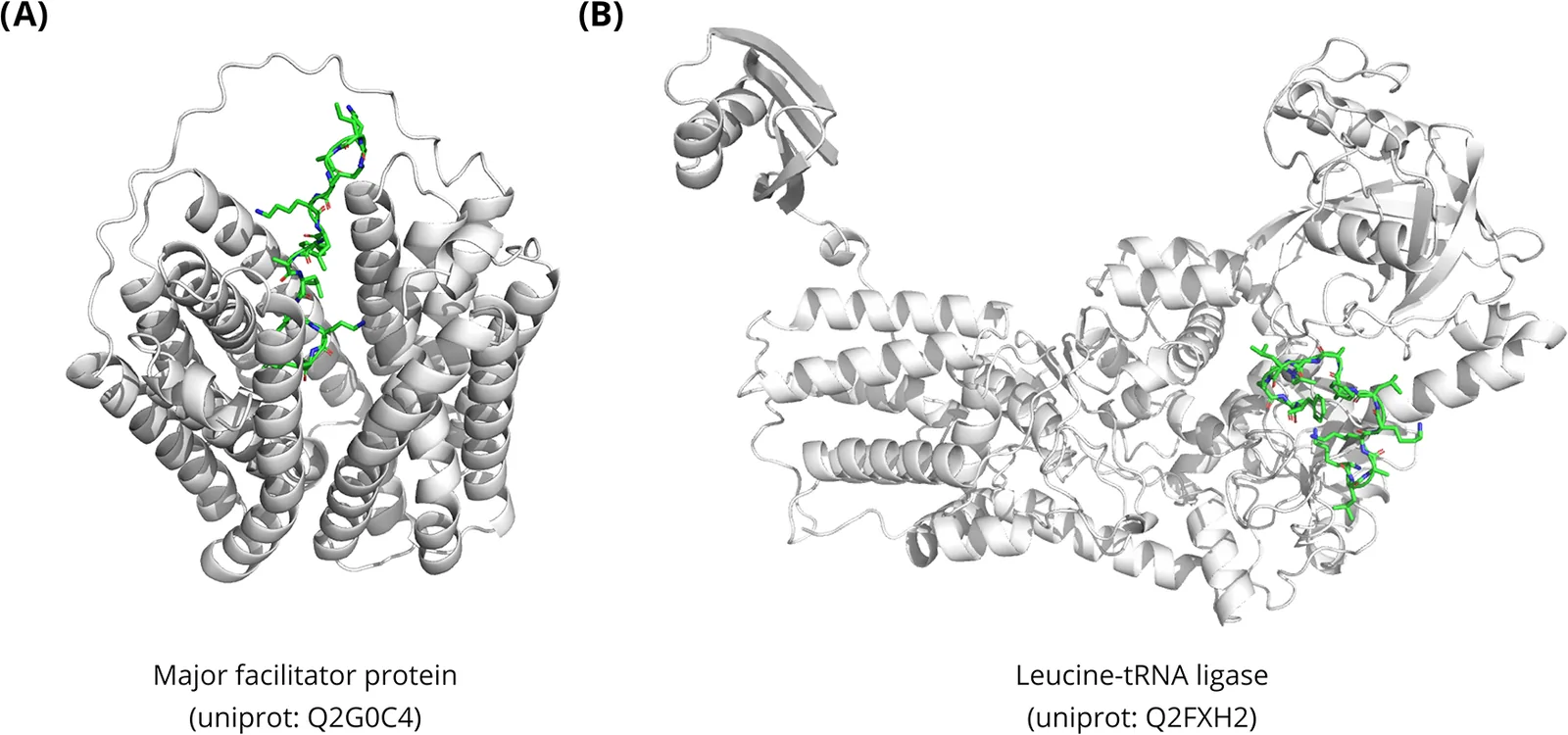
Potential targets of PEP-4 (rendered in green) on the *Staphylococcus aureus* proteome (rendered in gray) identified via *in silico* reverse docking. (A) Complexation with the membrane-embedded Major Facilitator Superfamily (MFS) transporter (Q2G0C4; Δ*G* = −59.24 kcal/mol), suggesting a potential interference with transmembrane transport. (B) Intracellular binding to Leucine-tRNA ligase (Q2FXH2; Δ*G* = −56.18 kcal/mol).

**Figure 2:**
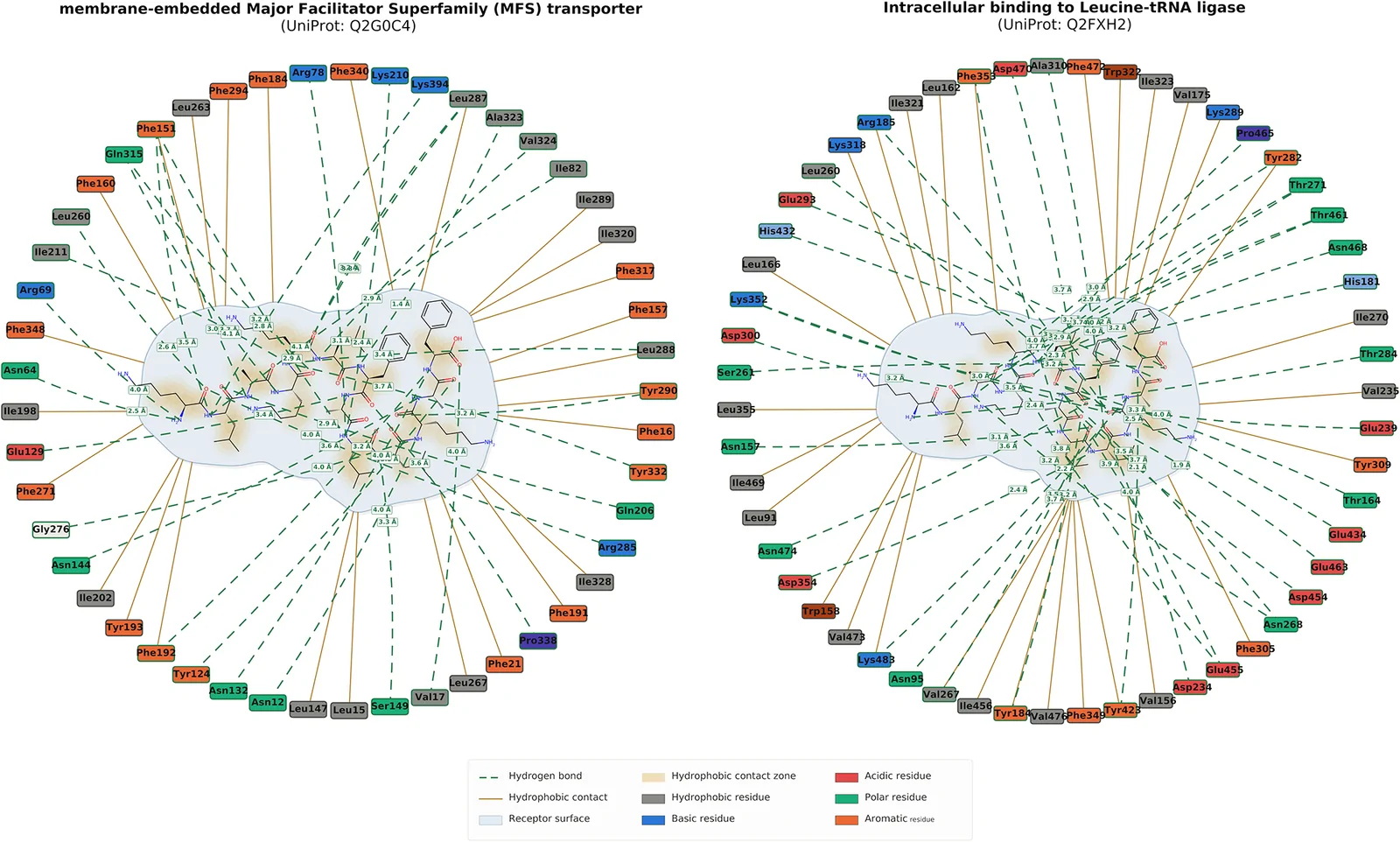
2D representation of the residues involved in the interaction between the antimicrobial peptide PEP-4 and predicted target proteins of *Staphylococcus aureus*, the membrane-embedded Major Facilitator Superfamily (MFS) transporter (Uniprot: Q2G0C4) and Leucine-tRNA ligase (Uniprot: Q2FXH2). The legend highlights key structural interactions, including hydrogen bonds, hydrophobic contacts, and hydrophobic contact zones, as well as residue classifications (acidic, basic, polar, hydrophobic, and aromatic).

**Table 5:** Protein-Peptide Affinity of PEP-4 and the Proteins in the *Staphylococcus aureus* Proteome Predicted Using AutoDock CrankPeptide (ADCP) Based on Structures Predicted by AlphaFold

Uniprot ID	binding energy (kcal/mol)	protein name	GO terms
Q2G0C4	–59.24	major facilitator protein	plasma membrane (GO:0005886)
			transmembrane transporter activity (GO:0022857)
			transmembrane transport (GO:0055085)
Q2FXH2	–56.18	leucine-tRNA ligase	cytosol (GO:0005829)
			aminoacyl-tRNA deacylase activity (GO:0002161)
			ATP binding (GO:0005524)
			leucine-tRNA ligase activity (GO:0004823)
			leucyl-tRNA aminoacylation (GO:0006429)
Q2FV37	–54.38	nucleoside transporter/FeoB GTPase gate domain-containing protein	
Q2FZN1	–51.96	YxeA family protein	
Q2FXF3	–51.80	Staphylococcal protein	
Q2FXY4	–51.05	DNA internalization-related competence protein ComEC/Rec2	plasma membrane (GO:0005886)
			establishment of competence for transformation (GO:0030420)
Q2FXC0	–50.17	DUF4888 domain-containing protein	
Q2FYN0	–48.49	DUF1033 family protein	
Q2FZP6	–48.44	UDP-N-acetylmuramoyl-l-alanyl-d-glutamate-l-lysine ligase	cytoplasm (GO:0005737)
			ATP binding (GO:0005524)
			ligase activity (GO:0016874)
			magnesium ion binding (GO:0000287)
			UDP-N-acetylmuramoyl-l-alanyl-d-glutamate-l-lysine ligase activity (GO:0047482)
			cell division (GO:0051301)
			cell wall organization (GO:0071555)
			peptidoglycan biosynthetic process (GO:0009252)
			regulation of cell shape (GO:0008360)
Q2FV19	–48.21	α-acetolactate decarboxylase	acetolactate decarboxylase activity (GO:0047605)
			acetoin biosynthetic process (GO:0045151)
Q2G0L8	–47.94	Cof-type HAD-IIB family hydrolase	cytosol (GO:0005829)
			magnesium ion binding (GO:0000287)
			phosphatase activity (GO:0016791)
2G0M7	–46.25	protein/nucleic acid deglycase HchA	cytoplasm (GO:0005737)
			glyoxalase III activity (GO:0019172)
			protein deglycase activity (GO:0036524)
			thiolester hydrolase activity (GO:0016790)
			DNA repair (GO:0006281)
			methylglyoxal catabolic process to d-lactate via S-lactoyl-glutathione (GO:0019243)
			protein repair (GO:0030091)
Q2FXC1	–45.70	uncharacterized protein	
Q2FXA3	–45.25	uroporphyrinogen decarboxylase	cytosol (GO:0005829)
			uroporphyrinogen decarboxylase activity (GO:0004853)
			heme biosynthetic process (GO:0006783)
			protoporphyrinogen IX biosynthetic process (GO:0006782)
Q2FY54	–45.24	dihydrolipoamide acetyltransferase component of pyruvate dehydrogenase complex	cytosol (GO:0005829)
			dihydrolipoyllysine-residue acetyltransferase activity (GO:0004742) dihydrolipoyllysine-residue succinyltransferase activity (GO:0004149)
			tricarboxylic acid cycle (GO:0006099)
Q2FXY0	–45.17	CP-type G domain-containing protein	GTP binding (GO:0005525)
Q2FZ16	–44.90	peptidase M16 C-terminal domain-containing protein	metal ion binding (GO:0046872)
Q2G108	–44.67	integrase	DNA binding (GO:0003677)
			DNA integration (GO:0015074)
			DNA recombination (GO:0006310)
Q2FZU3	–44.38	GP-PDE domain-containing protein	phosphoric diester hydrolase activity (GO:0008081)
			lipid metabolic process (GO:0006629)
Q2FVP2	–44.36	magnesium transporter CorA family protein	membrane (GO:0016020)
			metal ion transmembrane transporter activity (GO:0046873)

**Table 6:** Residues Involved in the Interaction between the Antimicrobial Peptide PEP-4 and Predicted Target Proteins of *Staphylococcus aureus*, the Membrane-Embedded Major Facilitator Superfamily (MFS) (Uniprot: Q2G0C4) and Intracellular Binding to Leucine-tRNA Ligase (Uniprot: Q2FXH2)

Uniprot ID	protein name	binding site residues
Q2G0C4	membrane-embedded Major Facilitator Superfamily (MFS) transporter	Ala1, Met2, Ala6, Ser7, Ile8, Ala11, Ser12, Ser13, Asn14, Leu15, Phe16, Val18, Lys19, His20, Lys21, Leu22, Tyr25, Leu26, Met27, Leu28, Gly29, Tyr30, Leu31, Ile32, Gln33, Pro34, Met38, Phe39
Q2FXH2	Intracellular binding to leucine-tRNA ligase	Gln8, Thr9, Phe10, Glu15, Val16, Ser17, Val18, Gln19, Asn23, Val24, Val25, His26, Tyr27, Lys28, Arg29, Val30, Val31, Trp32, Gly33, Phe34, Lys35, Leu41, Gln42, Ile43, Ile45, Leu46, Phe47, Gly48, Thr49, Ser58

As shown in Figure 3, the interactions of PEP-4 with the bacterial membrane model (POPG/DPPGK) and the mammalian membrane model (DOPC) were evaluated over a 1 μs simulation trajectory. Minimum distance analysis confirmed rapid adsorption in both systems. PEP-4 made direct contact with the lipid bilayers, reaching a stable minimum distance of approximately 0.18 nm. Despite identical minimum contact distances, the binding affinity exhibited strict membrane selectivity. Interfacial contact analysis (0.35 nm threshold) showed a significantly higher and more sustained number of atomic contacts between PEP-4 and the bacterial membrane model (POPG/DPPGK) than with the mammalian membrane model (DOPC). This indicates strong electrostatic accumulation on the bacterial membrane, whereas interactions with the mammalian model were superficial and uncoordinated.

**Figure 3:**
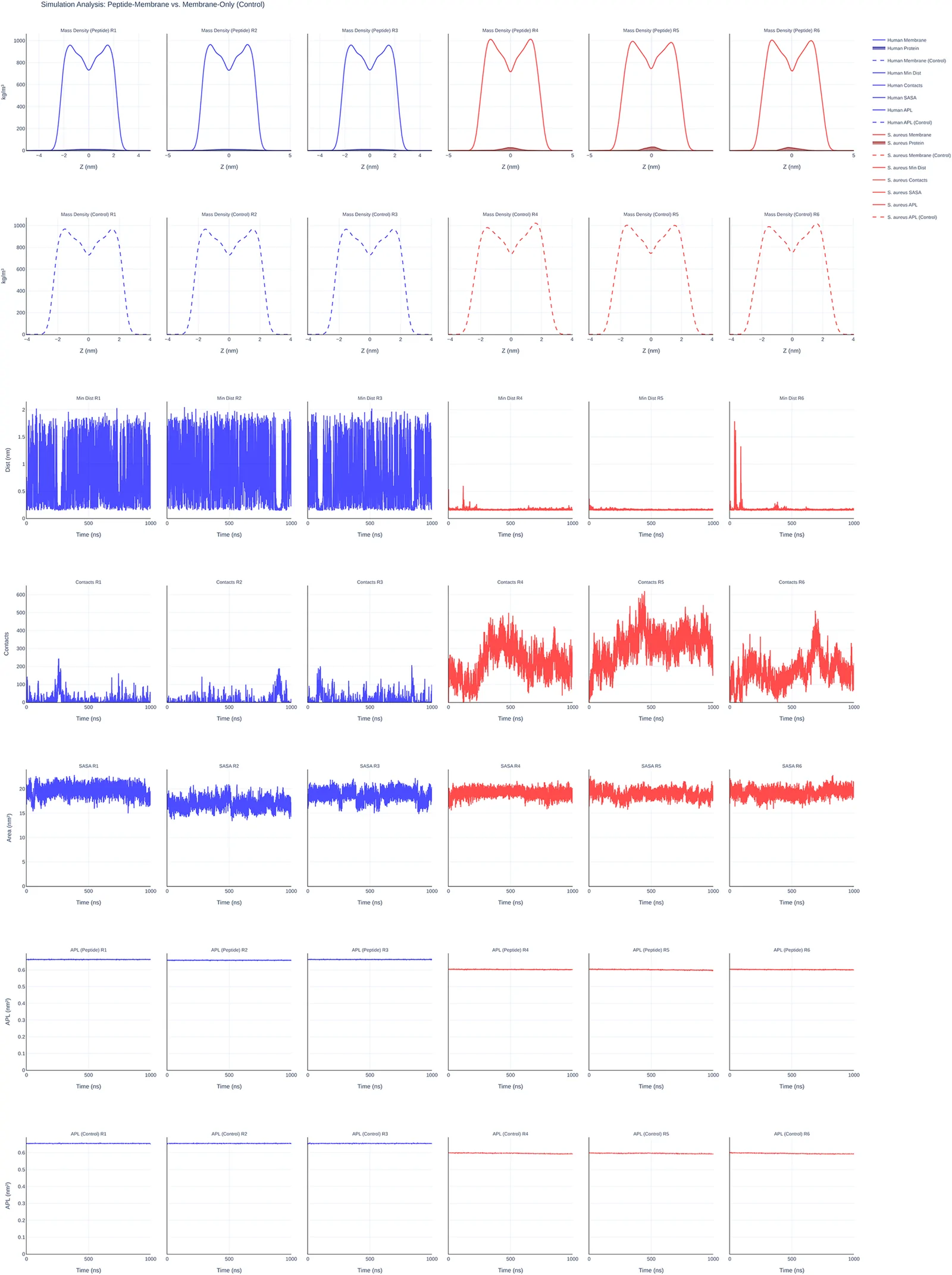
Molecular dynamics simulation of PEP-4 interacting with bacterial membrane model (POPG/DPPGK; red) and mammalian membrane model (DOPC; blue) across three independent 1 μs trajectories. *Z*-axis mass density profiles map peptide partitioning relative to the lipid bilayer boundaries, showing persistent surface association in the headgroup region of the bacterial membrane model (POPG/DPPGK), whereas interactions with the mammalian membrane model (DOPC) remain highly transient at the solvent interface. The minimum distance between the peptide and the lipid bilayer establishes the timeline of surface adsorption. Interfacial atomic contacts (threshold <0.35 nm) quantify the differential binding affinity, with sustained contact on the bacterial membrane model and intermittent contact on the mammalian model. Total membrane Area and Area Per Lipid (APL) trajectories remain stable and indistinguishable from the corresponding membrane-only controls, indicating no detectable perturbation of lipid packing in either system over the simulated time frame.

*Z*-axis mass density profiles were used to compare the spatial distribution of PEP-4 relative to that of the bilayer center in the two systems. In the POPG/DPPGK system, the peptide density was distributed across the bilayer interface, consistent with the sustained contact observed in the minimum distance and contact analyses. In the DOPC system, the peptide density was negligible over the same range, reflecting the intermittent nature of its association with the zwitterionic bilayer. In both systems, the lipid density profile was superimposable on the corresponding membrane-only control, indicating that peptide association did not measurably alter the transverse organization of either bilayer. Because a single peptide contributes a small fraction of the total system mass, these profiles were not used to assign a specific insertion depth.

Lateral box dimensions (X and Y vectors) were monitored to derive the Area Per Lipid (APL) and were compared with peptide-free control simulations for each bilayer. Both the POPG/DPPGK and DOPC systems maintained stable lateral dimensions over the full 1 μs, and the APL values of the peptide-containing systems were indistinguishable from their respective controls. Under the conditions simulated here, a single peptide per bilayer over 1 μs, PEP-4 association did not produce a detectable change in lipid packing in either membrane. This is consistent with threshold-dependent models of AMP activity, in which bilayer destabilization requires accumulation of multiple peptides at the membrane surface, a regime that single-peptide simulations cannot sample.

## Discussion

Machine learning algorithms and regression models predicted the synthetic peptides’ baseline nontoxicity, nonhemolytic activity, and general antibacterial activity. However, these tools failed to resolve the nuances of the critical sequence-activity relationship (SAR). Uniform classification as “Active” masked the significant biological variance we observed *in vitro*, where permutations of the C-terminal residues shifted MIC values by an order of magnitude. Computational filtering serves exclusively as a primary gating mechanism and lacks the resolution to predict the precise membrane partitioning dynamics that dictate absolute potency.

Generative models trained on AMP databases inherently reproduce established α-helical amphipathic motifs. While our sequences share structural homology and conserved core physicochemical characteristics, the spatial orientation and composition of the C-terminal hydrophobic residues are the primary determinants of efficacy. PEP-4 (terminal sequence: KLF) combines a low normalized hydrophobicity with an optimized insertion tilt angle and penetration depth. The C-terminal Leucine–Phenylalanine motif is associated with the highest *in vitro* potency in the series and, in the simulations, with persistent membrane association, while distributed lysine residues promote initial electrostatic recruitment to anionic membranes.

PEP-4 demonstrates distinct susceptibility profiles delineated by bacterial cell envelope architecture. Against all tested Gram-negative ESKAPE pathogens (*Klebsiella pneumoniae*, *A. baumannii*, *P. aeruginosa*, *K. aerogenes*), PEP-4 maintains an exact 1:1 MBC/MIC ratio (62.5 μg mL^–1^). The observation that MIC and MBC values are identical for several Gram-negative species indicates that bacterial growth inhibition and bactericidal activity occur within a narrow concentration range. However, the present data do not allow direct inference of the underlying killing mechanism, which would require dedicated membrane permeability and time-kill experiments.

The differential susceptibility observed among the tested ESKAPE pathogens is likely attributable to fundamental differences in the cell envelope architecture. Gram-positive bacteria lack the outer membrane and lipopolysaccharide (LPS) barrier that is characteristic of Gram-negative organisms. The absence of this additional permeability barrier facilitates the access of amphipathic peptides to the cytoplasmic membrane, which may contribute to the lower MIC values observed against Gram-positive species in the present study. In contrast, the outer membrane of Gram-negative bacteria contains a densely packed LPS layer that functions as both a physical and an electrostatic barrier, limiting the penetration of many antimicrobial compounds and AMPs. This additional envelope complexity likely contributes to the reduced susceptibility of Gram-negative species and the higher peptide concentrations required to achieve comparable antibacterial effects. Because PEP-4 is a cationic amphipathic peptide, its interaction with the bacterial surface is expected to be strongly influenced by these structural differences, potentially explaining the variation in the antimicrobial activity observed among the tested pathogens.

In this context, PEP-4 seems to be the most promising lead peptide. It unites the optimal predicted safety profile with superior broad-spectrum bactericidal activity. The gap between bactericidal concentrations (31.2–62.5 μg mL^–1^, corresponding to ∼20.9–41.9 μM) and the predicted hemolytic threshold (HC50 = 234.590 μM) suggests a promising therapeutic window, pending *in vitro* cytotoxicity validation. We retain PEP-1 as a secondary peptide scaffold due to its moderate broad-spectrum activity. We eliminate PEP-2, PEP-3, and PEP-5 from further analysis due to subtherapeutic potency profiles.

The divergent binding kinetics observed over the 1 μs trajectories are consistent with the proposed physicochemical basis for PEP-4 selectivity. The mammalian membrane model (DOPC), composed exclusively of zwitterionic lipids, lacks sufficient electronegative surface potential to recruit the cationic peptide. Consequently, the absence of stable electrostatic anchoring is consistent with the intermittent association observed in this system, and with the predicted lack of cytotoxicity and hemolytic activity.^28^

In contrast, the bacterial membrane model (POPG/DPPGK) incorporates anionic phosphatidylglycerol (POPG) alongside lysylated derivatives (DPPGK), reproducing the charge-modulated bacterial envelope of *Staphylococcus aureus*. Although lysyl-phosphatidylglycerol (Lys-PG) reduces the net negative charge and attenuates AMP binding as part of a well-characterized resistance mechanism, it does not fully abrogate peptide–membrane interactions.^29^ The sustained proximity of PEP-4 to this bilayer indicates its ability to overcome this electrostatic barrier, likely through preferential binding to residual anionic POPG domains. Initial electrostatic anchoring to these domains is the step captured by the present simulations. In the canonical amphipathic AMP mechanism, this anchoring precedes hydrophobic partitioning of the apolar face into the acyl-chain region; the correlation between C-terminal hydrophobicity and *in vitro* potency across the peptide series is consistent with such a step contributing to activity, but the present simulations neither resolve the depth of the Leucine–Phenylalanine motif nor detect a change in acyl chain packing, and the insertion step therefore remains a hypothesis. The sequential electrostatic–hydrophobic mechanism is a defining feature of amphipathic AMPs, whereby cationic residues mediate membrane recognition and hydrophobic regions drive insertion and disruption.^30^

Threshold-dependent models of AMP activity hold that bilayer perturbation requires cooperative interactions among multiple peptides once a critical surface concentration is reached.^31^ The single-peptide systems simulated here cannot access this regime, which is the most likely reason that no perturbation was detected. Multipeptide simulations would be required to test whether PEP-4 reaches such a threshold on the *S. aureus* bilayer. What the present simulations establish is the preceding and necessary step: PEP-4 is selectively and persistently recruited to the anionic, Lys-PG-containing bilayer and not to the zwitterionic mammalian model.

The antimicrobial activity of PEP-4 involves preferential interactions with bacterial membranes, resulting in a persistent and selective association at the membrane interface. Beyond membrane association, reverse docking identified potential secondary interaction partners in *S. aureus*, such as the membrane-embedded major facilitator superfamily (MFS) transporter (Q2G0C4), which could impair transmembrane transport and efflux pathways.^32,33^

The binding affinity observed for leucine-tRNA ligase (Q2FXH2) is relevant given the growing interest in antimicrobials targeting tRNA ligases.^34,35^ In the case of peptides, at sublethal local concentrations, or during the transient phase preceding the attainment of the critical threshold required for catastrophic membrane disruption, a fraction of translocated peptide molecules may engage intracellular targets and interact with proteins involved in translation-related processes.^36^

Inhibition of aminoacyl-tRNA synthetases represents a plausible complementary inhibitory axis, as disruption of these enzymes directly compromises translational flux and bacterial envelope stress responses.^37,38^

While these integrated *in silico* and *in vitro* methodologies establish a structural framework for predicting the mechanistic behavior and safety profile of PEP-4, several key limitations of the current study must be noted. First, hemolysis and mammalian cytotoxicity assays were not performed by the authors, meaning safety assessments and HC50 values remain based on computational predictions rather than experimental validation. Second, the computational membrane models employ simplified lipid compositions that do not fully capture the structural complexity and diversity of native bacterial or mammalian bilayers. Third, reverse docking relies on static predicted protein structures from AlphaFold, which may not account for conformational flexibility or physiological binding states. Fourth, the microsecond time scale of molecular dynamics simulations, while extensive, cannot model large-scale membrane pore formation or long-term structural changes. In addition, the molecular dynamics analyses reported here characterize peptide–membrane association rather than insertion or permeabilization. Residue-resolved insertion depth, lipid acyl-chain order parameters, bilayer thickness, and water defect analysis were not computed, and multipeptide systems were not simulated; consequently, the insertion and disruption steps of the proposed mechanism remain untested. Finally, these computational predictions require empirical validation through downstream *in vitro* cytotoxicity and target-binding assays.

## Conclusion

The rapid emergence of antimicrobial resistance among ESKAPE pathogens necessitates the development of next-generation therapeutics with novel mechanisms of action. Here, we present an integrated *in vitro* and *in silico* evaluation of rationally designed synthetic peptides, identifying PEP-4 as a lead peptide with potent and selective bactericidal activity. Despite comparable sequence-derived physicochemical properties, conventional predictive models failed to capture the critical contribution of C-terminal hydrophobic optimization to functional potency. PEP-4, characterized by a Leucine–Phenylalanine terminal motif, exhibited broad-spectrum activity (MIC 15.6–62.5 μg mL^–1^, corresponding to ∼10.5–41.9 μM). We observed notable activity against Gram-positive pathogens, including *Staphylococcus aureus* (MIC 15.6 μg mL^–1^, ∼10.5 μM) and *Enterococcus faecium* (MIC 31.2 μg mL^–1^, ∼20.9 μM), achieving bactericidal activity at 2-fold higher concentrations. We also observed consistent bactericidal activity against Gram-negative pathogens, with 1:1 MBC/MIC ratios.

Microsecond-scale molecular dynamics simulations revealed persistent membrane association and greater lipid contact with bacterial membrane models than with zwitterionic mammalian models. Reverse docking further identified potential intracellular targets, such as leucyl-tRNA ligase and MFS transporters, as plausible secondary mechanisms.

In contrast, association with the mammalian membrane model (DOPC) was intermittent throughout all three replicates, with the peptide repeatedly detaching from the interface. This difference in binding residence time and interfacial contact number provides a charge-based mechanistic basis for the selectivity predicted by the in silico safety profile. This divergence is reflected experimentally by low cytotoxicity (HC_50_ = 234.590 μM), defining a predicted therapeutic window. Collectively, these findings identify PEP-4 as a promising peptide scaffold and highlight the importance of C-terminal hydrophobic optimization in governing peptide–membrane interactions. This work provides both a promising lead peptide and a mechanistically informed framework for the rational design of next-generation AMPs.
